# B Chromosomes’ Sequences in Yellow-Necked Mice *Apodemus flavicollis*—Exploring the Transcription

**DOI:** 10.3390/life12010050

**Published:** 2021-12-30

**Authors:** Marija Rajičić, Alexey Makunin, Tanja Adnađević, Vladimir Trifonov, Mladen Vujošević, Jelena Blagojević

**Affiliations:** 1 Department of Genetic Research, Institute for Biological Research “Siniša Stanković”, National Institute of Republic of Serbia, University of Belgrade, Bulevar Despota Stefana 142, 11000 Belgrade, Serbia; tanja.adnadjevic@ibiss.bg.ac.rs (T.A.); mladenvu@ibiss.bg.ac.rs (M.V.); jelena.blagojevic@ibiss.bg.ac.rs (J.B.); 2 Department of the Diversity and Evolution of Genomes, Institute of Molecular and Cellular Biology, Siberian Branch of the Russian Academy of Sciences, 630090 Novosibirsk, Russia; alex@mcb.nsc.ru (A.M.); vlad@mcb.nsc.ru (V.T.)

**Keywords:** B chromosomes, RT-PCR, mRNA, *Apodemus flavicollis*

## Abstract

B chromosomes (Bs) are highly polymorphic additional chromosomes in the genomes of many species. Due to the dispensability of Bs and the lack of noticeable phenotypic effects in their carriers, they were considered genetically inert for a long time. Recent studies on Bs in *Apodemus flavicollis* revealed their genetic composition, potential origin, and spatial organization in the interphase nucleus. Surprisingly, the genetic content of Bs in this species is preserved in all studied samples, even in geographically distinct populations, indicating its biological importance. Using RT-PCR we studied the transcription activity of three genes (*Rraga*, *Haus6*, and *Cenpe*) previously identified on Bs in *A. flavicollis*. We analysed mRNA isolated from spleen tissues of 34 animals harboring different numbers of Bs (0–3).The products of transcriptional activity of the analysed sequences differ in individuals with and without Bs. We recorded B-genes and/or genes from the standard genome in the presence of Bs, showing sex-dependent higher levels of transcriptional activity. Furthermore, the transcriptional activity of *Cenpe* varied with the age of the animals differently in the group with and without Bs. With aging, the amount of product was only found to significantly decrease in B carriers. The potential biological significance of all these differences is discussed in the paper.

## 1. Introduction

B chromosomes (Bs) are dispensable karyotype elements, unnecessary for normal growth and development. Accordingly, they are expected to accumulate mutations and harbor pseudogenes [1]. B chromosomes prove to be so challenging for research because they constitute the most widespread chromosome polymorphism that includes a class of chromosomes that are tremendously variable concerning their morphology, structure, phenotypic effects, behaviour, origin, modes of transmission, and population maintenance. Different aspects of B chromosome biology are covered in the well-accepted reviews (among others, [2,3,4,5,6]). Together with the heterochromatic nature of many Bs, those additional members of a karyotype have been considered as non-active with no contribution to the transcriptome for decades [3]. 

Besides using classical cytogenetic and molecular-cytogenetic methods for investigating Bs in general, modern studies of Bs’ sequences take advantage of next-generation-sequencing (NGS), transcriptomic analysis followed by analytical methodologies. This has helped to develop a better understanding of the origin, molecular structure, transcriptional status, potential biological role, and evolution of these additional karyotype elements.

Even before direct evidence of the transcription activity of Bs’ sequences appeared, there was indirect evidence that difference in expression of some genes is correlated with the presence of Bs in disparate species [7,8,9,10,11].

Identification of genes present on Bs through molecular-cytogenetic methods is summarised for mammals in Vujošević et al. [12], and the increasing number of sequenced B chromosomes [13,14], has permitted studies on transcriptional activity of repetitive, protein-coding sequences, pseudogenes located on Bs along with B-specific sequences. Transcription of Bs sequences is confirmed in different species including several plants: the smooth hawksbeard [15], rye [16,17], and maize [18]. B-associated transcripts were also identified in parasitic worms [19], grasshoppers [20,21], cichlid fish [13], and Siberian roe deer [22].

Furthermore, transcriptomics enables a global overview of Bs’ influence on the activity of the whole genome by comparing genomes with and without Bs. Comparative studies of complete transcriptomes between individuals with and without Bs indicate that Bs influence cell biology in a complex manner [23]. Different studies proposed that Bs influence the main genome expression through noncoding RNA [18,24,25,26,27,28,29]. Likewise, it was shown that some of Bs protein-coding genes were, not only transcriptionally active, but also significantly up-regulated in B-carrying individuals [21]. In addition, the first gene on Bs, coding a functional protein in vitro, was identified [17].

Expression of Bs sequences in some species is correlated with the female sex. This is the case in fish, *Astatolapia latifasciata* [13], and in other cichlids also [14,30]. This connection between Bs and the female sex could indicate an increase in the adaptive value in female carriers of Bs [31].

On the territory of Serbia, Bs in the yellow-necked mice, *Apodemus flavicollis*, were found in more than 30 populations in a wide range of frequencies from 7% to 64% [32,33,34]. The highest number of Bs detected in Serbia was five [35]. Furthermore, there is no recorded difference in the number and frequency of Bs between male and female carriers [34,36,37]. In this species, numerous population studies have shown that a higher percentage of B carriers is present in environmental conditions that are not optimal for this species. So, the frequency of Bs increases with altitude and is positively correlated with the average number of sub-zero days but negatively with average temperature [34]. Exceptions to this general trend are noted in Poland [38]. Equilibrium frequencies of Bs were found during the course of 5 years’ study, in spite of changes in population density [36], but it was found that seasonal changes in the frequency of Bs could be significant in the presence overcrowding [39]. Furthermore, different morphometric studies have highlighted that numerous phenotype characteristics are correlated with the presence of Bs [11,37,40,41]. The mentioned data suggest that Bs in *A. flavicollis* show effects at the population level [34,37,42], and that in specific environmental conditions Bs could be beneficial for their carriers [39,43,44].

Microdissection of Bs, together with their molecular-cytogenetic analysis, indicated the origin of the additional karyotypic element in *A. flavicollis* from the pericentromeric region (PR) of sex chromosomes [45,46]. Furthermore, an NGS analysis of micro-dissected Bs and comparison of obtained sequences to the referent genome of house mouse, *Mus musculus*, allowed identification of 22 chromosomal regions that originate from sixteen chromosomes of the reference genome, thus depicting the Bs in this species as mosaics of multichromosomal origin. The analysis identified 39 protein-coding genes on Bs in *A. flavicollis*, and the majority of these were microtubule and cell-cycle-associated genes. Some of these genes are at different stages of decay and others are disrupted by multiple missense substitutions, making them B-specific. The same study also demonstrates that all analyzed Bs were composed of the same A chromosomes regions. Only one population-specific region was identified in a sample from Russia [47]. This observation and earlier studies of structure [48], suggest that Bs in this species are somehow structurally conserved. Molecular content preserved in this way could be the result of the functional role that Bs gained through their evolution in *A. flavicollis*.

With the aim to better understand Bs structure and role we conducted a study on the transcription activity of three sequences identified on Bs in *A. flavicollis* depending on the presence of Bs and age of the animal.

## 2. Materials and Methods

### 2.1. Samples

This study included RNA samples isolated from 34 specimens: 14 without Bs, 14 with one, five with two, and one with three Bs (Table 1). The animals used in this study were collected from a natural populations using Longworth traps and were treated according to legal and ethical guidelines as indicated in Directive 2010/63/ EU of the European Parliament and Council of 22 September 2010 on the protection of animals used for scientific purposes. A dry eyes lens mass was used as the best indicator of the samples’ age [49]. The specimens we chose for this study were trapped in 14 different localities and were mostly young individuals (dry eyes lens mass less than 22 mg) and equally represented both sexes within the groups: the group with Bs and the group without Bs.

Chromosomes preparation was done from bone marrow (Hsu and Patton 1969). To determine the number of Bs in karyotype, 20 metaphase plates were counted per animal.

### 2.2. RNA and DNA Extraction

We conducted a study on RNA and DNA extracted from samples listed in Table 1. To eliminate tissue and age specificity in a transcription analysis of B sequences, we used total RNA and DNA isolated from the same piece of spleen tissue of young individuals.

The total RNA was isolated from spleen tissue using TRIzol^®^ Reagent (Thermo Fisher Scientific, Waltham, MA, USA), according to the following protocol. Approximately 20 mg of tissue was homogenized in the presence of liquid nitrogen; 1 mL of TRIzol Reagent was added and incubated at room temperature. A further 200 µL of chloroform per mL of TRIzol was added and vortexed for 15 s. Then, incubation at room temperature for 15 min with a periodical vortex was performed, and finally the treated tissue was centrifuged (12,000× *g*, 4 °C, 15 min). The supernatant, for which the content of total RNA was separated and gently mixed with 500 µL of isopropyl alcohol and incubated at room temperature for 10 min and centrifuged after that (12,000× *g*, 4 °C, 15 min). Lower phases were stored at −20 °C and used for DNA isolation. RNA became visible as white sediment. The supernatant was poured off and the sediment gently washed with 75% ice-cold ethanol. After one more centrifuge (12,000× *g*, 4 °C, 15 min) the supernatant was cast, and the sediment was dried at room temperature. The RNA sediment was dissolved in 50 µL of diethyl pyro carbonate treated (DEPC) water. According to the producer’s protocol the possible presence of DNA was eliminated by using the DNase I enzyme (Thermo Fisher Scientific, Waltham, MA, USA).

DNA was extracted from the previously saved, lower phases of solutions obtained and stored at −20 °C during RNA extraction. Firstly, the remaining amount of RNA supernatant was carefully removed. Then, 100% of ethanol was added in 3.3 times less volume than the TRIzol at the beginning. After incubation at room temperature for 2–3 min and centrifuge (2000× *g*, 4 °C, 5 min), the supernatant was removed, and DNA residue was washed two times in the solution: 1 M trisodium citrate in 10% ethanol. After each wash supernatant was removed. DNA precipitate was dissolved in 75% of ethanol 400 µL, incubation at room temperature for 5 min with a periodical shaking, and centrifuge at the end (12,000× *g*, 8 °C, 5 min). Ethanol was removed and DNA precipitate was dried at room temperature for 5 min. DNA was dissolved in 60 µL of 8 mM NaOH and centrifuged once again (12,000× *g*, 10 min). The supernatant containing DNA was transfer to the new tubes.

The concentration of RNA and DNA was measured by NanoPhotometer N60 Touch (Implen, München, Germany).

### 2.3. Primer Design

This study is based on known B sequences provided in former studies on Bs in *A. flavicollis* [45,47]. In order to detect transcription that originates from B in this species, we searched through the B sequences for the regions where pseudogenisation largely occurred in comparison with the reference genome. We used variant calls from combined an *A. flavicollis* B chromosome library aligned to a mouse reference genome (mm10) [47]. Variant effects were annotated based on RefSeq gene annotation for mm10 [47]. B-chromosome specific fasta files were generated with GATK v.3.4 FastaAlternateReferenceMaker for exons located inside genomic regions found on Bs (region detection also described in [47]). Furthermore, we examined vcf for the disruptive and/or high density mutations and used the B-specific fasta for primer design. Due to the incomplete genomic coverage of chromosome-specific libraries, only a handful of sequences were shortlisted for primer design. This way, we selected the following sequence regions as candidates for primer pairs: *Cenpe* (multiple missense substitutions in exon 27, highly differentiated exon 28), *Rraga* (frameshift insertion in exon 1), *Kdm6a* (stop gain in exon 16), *Haus6* (in-frame insertion in exon 16), *Ppp6r3* (two missense substitutions in exon1, two missense substitutions in exon16), *Dync1i2* (two missense substitutions in a single codon in exon 2, two missense substitutions in a single codon in exon 6). Primers were selected using online Primer3Plus [50]. Online software OligoAnalyser 3.1 (Integrated DNA Technologies Inc., Coralville, USA) was used to estimate the primer efficiency. We obtained results connected with the presence of Bs and detectable by the PCR using DNA as a template for only three tested primer pairs (*Rraga*, *Haus6*, and *Cenpe*) (listed in Table 2).

### 2.4. PCR

Firstly, we studied the presence of three target sequences (*Rraga*, *Haus6*, and *Cenpe*) in all samples involved in study, using DNA as a template. In this way, we avoided false-negative results deriving from tissue mosaicism and the potential absence of Bs in spleen tissue, or the inconsistent presence of those sequences in different Bs. The reaction was carried out in 1x PCR Gold Taq buffer, 1 μM dNTPs, 1 μM of each primer, and 1 U Gold Taq polymerase (Promega, Madison, WI, USA). We used the following cycling conditions: 95 °C, 2 min, 28 cycles of denaturation on 95 °C, 30 s, annealing 58 °C, 30 s, extension 72 °C, 45 s, and final extension 72 °C in 10 min. The reaction results were analyzed by electrophoresis in 1% agarose gel. A molecular marker (GeneRuler 100 bp DNA Ladder, Thermo Fisher Scientific, USD, Waltham, MA, USA) was used for determination of PCR product lengths.

### 2.5. RT-PCR

The RNA study was conducted after confirmation that the presence or amount of target sequences on DNA template was associated with the presence of Bs of all samples. We studied the presence and quantity of transcripts from three selected Bs sequences (*Rraga*, *Cenpe,* and *Haus6*) present in total RNA. The presence of target transcript was determined using melting curve.

The quantity of transcribed target sequences was determined by comparing the target gene expression between group with Bs and a group without Bs as a control group, using reference genes (*Calnexin*, *Pgk*, and *β-actin*) (listed in Table 2), as a standard. For quantification, we used the Ct comparative method RQ = 2^−ddCt^. The melting temperature was used to detect the specificity of reaction products.

The polymerase chain reaction in real-time (RT-PCR) was performed on AB Prism 7000 Sequence Detector System (Applied Biosystems, Waltham, MA, USA), using KAPA tm SYBR FAST One-Step qRT-PCR Kit (Kapa Biosystems, Boston, MA, USA). Reactions were performed in a volume of 10 μL which contained KAPA SYBR FAST qPCR Master Mix (1×), (1 μM) primers, dUTP (10 mM), ROX High, Kapa RT Mix (1×), and RNA as a template. The temperature profile of the reaction was according to the producer’s recommendation and included synthesis of cDNA at 42 °C for 5 min, inactivation of reverse transcriptase at 95 °C for 5 min, denaturation at 95 °C for 30 s followed by annealing, and extension at 58 °C or 60 °C for different primers for 1 min. The melting curve had automatically set a temperature profile of 95 °C, 15 s; 60 °C, 15 s, 95 °C, 15 s.

Results of the RT-PCR, representing relative concentrations of mRNA, were compared between groups of animals with (B+) and without Bs as a control group (B0), and also between sexes within groups with and without Bs by the Mann–Whitney U test in Statistica 7.0 (StatSoft Icn., Tulsa, OK, USA, 2004). The significance level was at *p* < 0.05. In the same program, we analysed the correlation between the mass of the dry eye lens (age) and relative concentrations of mRNA.

## 3. Results

**Figure 1:**
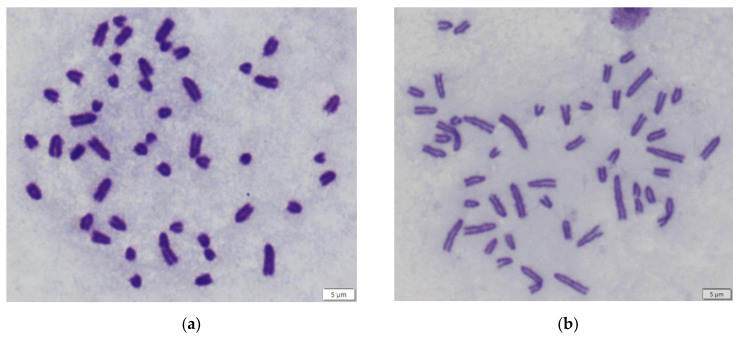
Metaphase figure of *Apodemus flavicollis* (**a**) animal 4134 from Table 1 (48,XY) (**b**) animal 4274 from Table 1 (48,XX,+1B) with all acrocentric chromosomes including B chromosome.

The most of analysed samples with B chromosomes were those with 1B (Figure 1).

**Figure 2:**
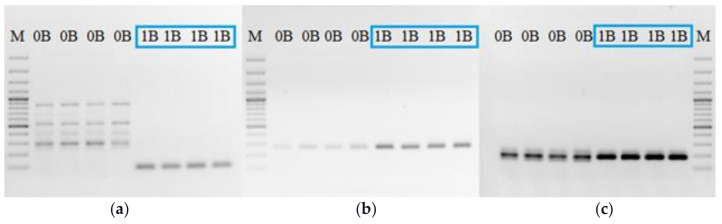
PCR products for genes: (**a**) Rraga; (**b**) Haus6; (**c**) Cenpe; DNA template from different samples with one B (1B) and without B (0B). M-DNA ladder 100 bp: (**a**,**b**) and 3000 bp (**c**).

For the study of Bs’ transcription activity we selected primer pairs based on known Bs sequences, considering the sequence difference between pseudogenes and original genes. The goal was to detect transcripts originating from Bs. The obtained PCR results on the DNA template, from samples with and without Bs, using three primer pairs, for *Rraga*, *Haus6*, and *Cenpe* sequences, indicate the overall difference in the presence of Bs (Figure 2).

The results for the *Rraga* gene showed a qualitative difference between samples with and without Bs, with a specific product in samples with Bs (Figure 2a). On the other hand, the primer pairs for *Haus6* (Figure 2b) and *Cenpe* (Figure 2c) showed that the quantitative product difference between samples with and without Bs. For the same starting concentration of DNA template, samples with Bs provided a much higher concentration of PCR product. Before the RT-PCR study, all studied samples were tested using DNA PCR test in order to avoid false negative results for example if some Bs did not contain a certain sequence.

**Figure 3:**
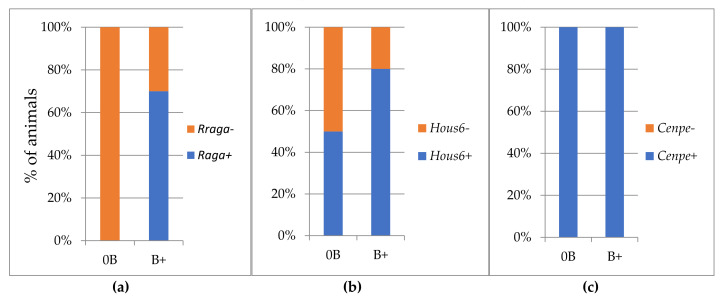
Percentage of animals with PCR products for genes: (**a**) *Rraga*; (**b**) *Haus6*; (**c**) *Cenpe*; without B (0B) and with B (B+).

The results of the RT-PCR product specificity for *Rraga* pseudogene primers correspond to the PCR results. In samples without Bs, nonspecific products with different melting temperatures were detected, while in samples with Bs in 70% of the analysed samples we recorded the occurrence of a product that melts at 79 °C (Figure 3a).

In the same way, *Haus6* transcript was detected in both groups, with and without Bs. Transcripts showed the same melting temperature, so we were not able to distinguish them regarding nucleotide content.

In any event, we recorded a difference between two groups in the percentage of samples in which *Haus6* transcript was present. The mRNA transcribed from the *Haus6* sequence was present in the group of animals without Bs in 50% of samples (Figure 3b), while in the B carriers, transcript was detected in 80% of samples.

The melting temperature did not indicate a difference in the sequence of *Cenpe* transcripts detected in groups with and without Bs, and mRNA was present in all analyzed samples in both groups (Figure 3c).

**Figure 4:**
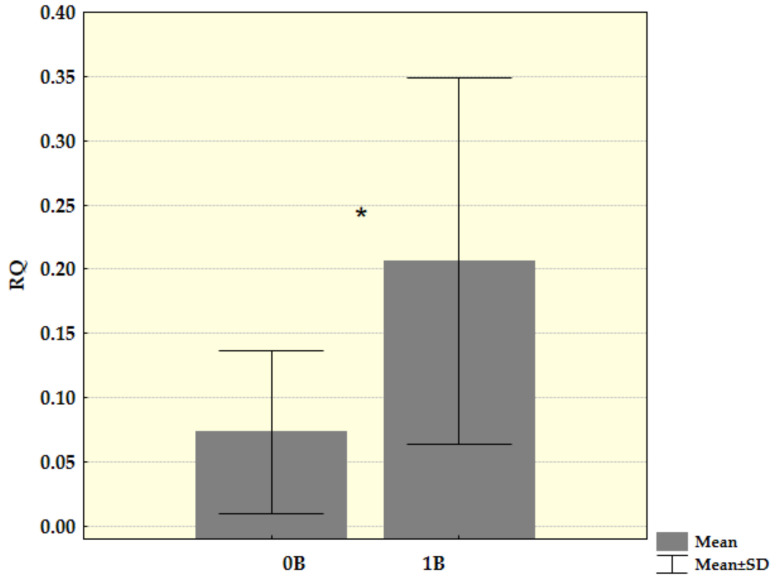
Relative mRNA quantification (RQ) for *Cenpe* gene in the spleen tissue of young samples divided into a group without B chromosomes (B0) and group with one B chromosomes (B1). Mean values and standard deviation (SD) for both groups were shown (* *p* < 0.05).

Since there was no difference in the presence and molecular structure of *Cenpe* transcripts, we could compare Ct values for these transcripts between groups of samples with and without Bs. Transcriptional activity was further analysed by the method of relative quantification. By comparing Ct values for *Cenpe* gene with reference genes and samples without B as control, we recorded a statistically significantly increased level of this gene transcription (Z = −2.149, *p* = 0.032) in Bs carriers. After the samples with more than one B chromosome were excluded from analysis, the difference in transcription level became more evident and stayed statistically significant (Z = −2.205, *p* = 0.027) (Figure 4).

**Figure 5:**
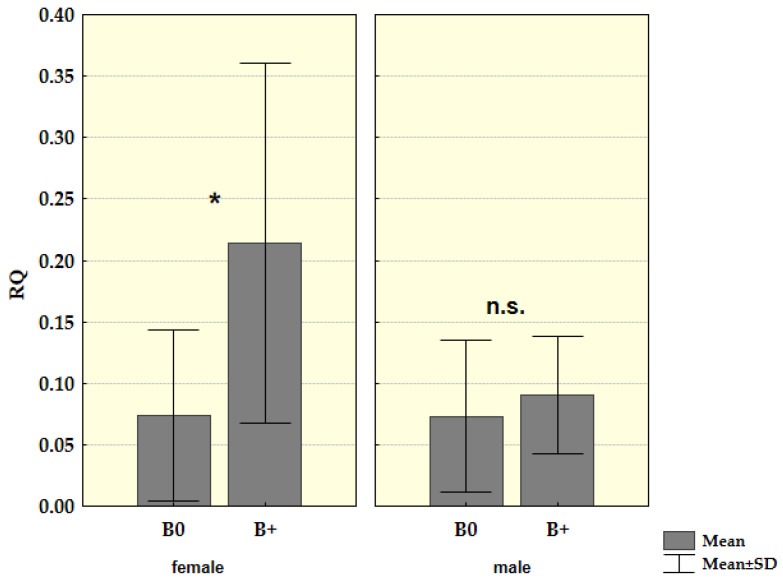
Relative mRNA quantification (RQ) for *Cenpe* gene in the spleen tissue of young male and female samples in a group without Bs (0B) and a group with one B chromosome (1B); * *p* < 0.05, n.s.—nonsignificant.

When we analyzed groups of males and females separately, males did not show a statistically significant difference (Z = −0.688, *p* = 0.491) in the presence of mRNA for *Cenpe* gene between B carriers and samples without Bs. On the other hand, in females, this difference was found to be statistically significant Z = −2.147, *p* = 0.032. Again, when we excluded samples with more than one B from analysis in the groups divided by sex, we obtained an even higher difference in females, Z = −2.364, *p* = 0.018, while in male groups there was still no significance Z = −0.703, *p* = 0.482 (Figure 5).

Keeping in mind that the samples we used in the analysis mainly originated from natural populations, besides Bs, the genetic background and environmental conditions were not controlled or even known. Consequently, we detected a large variability in the expression levels within the studied groups. The study highlighted the case of two young females from the same litter, with the same parents, grown under the identical laboratory conditions. The first female had one B chromosome (2*n* = 48, XX, +B) while the second had a standard complement (2*n* = 48, XX). Based on relative quantification (RQ) for *Cenpe* gene, the animal with 1B had 2.2 times more RNA products than its sister without a B chromosome. In this case, we consider that the effects of tissue specificity, sex and age differences and environmental factors were eliminated, and whole transcriptional activities could be assigned to B presence. Besides, the animal with one B also had transcripts of *Rraga*, and *Haus6* analysed sequences, while transcriptional activities were not detected in the animal without B. In summary, two genes showed a qualitative difference in expression, and one a quantitative difference in individuals from the same litter that differ in the presence/absence of B chromosomes in the genome.

**Figure 6:**
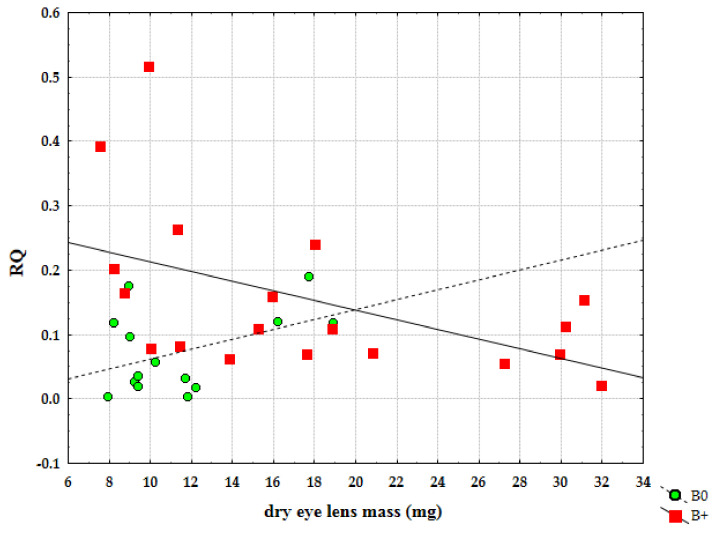
Correlation of relative quantification (RQ) for *Cenpe* gene and age between samples without B chromosomes (B0) and samples with Bs (B+).

Furthermore, we analysed the level of expression of *Cenpe* gene in relation to age. It was possible to notice that the expression of *Cenpe* gene statistically significantly decreased (Figure 6) with age in samples with Bs (r = −0.506, *p* = 0.027). On the other side, individuals without Bs showed an opposite trend of expression but with no significant statistical difference (r = 0.440, *p* = 0.116). The result in samples without Bs should be reconsidered since, in this group, we did not have the same percentage of older individuals. For the same reason, this analysis should be performed in more balanced groups with regard to age.

**Figure 7:**
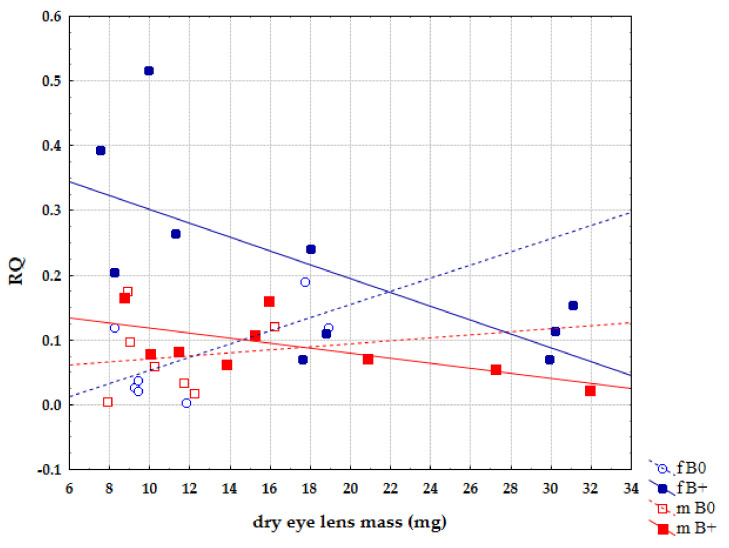
Correlation of relative quantification (RQ) for *Cenpe* gene and age between sexes in groups of samples without B chromosomes (B0) and samples with B chromosomes (B+).

The analysis of the separated sexes within groups of individuals with and without Bs showed that this trend of expression decrease is linked to the female sex, where it is statistically significant r = −0.678, *p* = 0.031 (Figure 7). In male groups, the same expression trends are present but there is no statistical significance.

## 4. Discussion

The prevalent heterochromatic appearance of Bs, revealed through cytogenetic studies [2,3], led to the long-lasting predominant view that Bs are transcriptionally inactive elements and “junk” DNA. Nevertheless, advances in molecular genetics have made it possible to destabilize this dogmatic view. Different studies of the noncoding DNA function, which present the majority of the eukaryotic genome [51], showed that noncoding DNA plays an important role in gene regulation and the organizational dynamic of the genome [52,53,54,55]. The main dogma of molecular biology that assumed genetic information to be mostly expressed through proteins, was expanded to RNA, since it was shown that a large part of genetic information is expressed through RNA levels [56]. Similar observations appeared regarding Bs structure and function in the genome [13].

The DNA analysis was used for screening the presence of sequences identified on B chromosomes for each sample. In all tested individuals with Bs from different populations in the territory of Serbia, we confirmed the presence of three tested B-specific sequences. This result confirms former studies suggesting that Bs in this species feature with stable molecular structure through the whole investigated distribution range [45,47,48]. This also supports the assumption that despite the rapid initial evolution of Bs, once they are established, their rate of further structural change and their accumulation of repetitive elements is greatly attenuated. In the various taxa, Bs evolved in similar ways, by accumulating sequences that are not random but are mostly genes involved in cell division [12].

By analyzing mutations and the accumulated differences between sequences located on Bs and their paralogs on A chromosomes, it is possible to detect if a particular sequence is transcribed from a standard complement (A) or B chromosome. In this way, the presence of a transcript enclosed by primers selected for B-specific sequence was recorded in 70% of B carriers’, while in the RNA samples without Bs it not been detected in the case of *Rraga* gene. *Rraga* gene encodes the Ras-related GTP-binding protein A that may play a crucial role in TNF-alpha signalling cascade, leading to induction of cell death [57]. Generally, it is known that around 20% of pseudogenes present in a standard genome are transcribed [58]. Additionally, it is shown that pseudogenes could affect the regulation of genes from which they originated [55]. Pseudogenes could regulate the expression of paralogs through ncRNA [59]. Furthermore, they could also act as sponges for miRNA [60]. Transcripts of pseudogenes could be translated into short peptides or even proteins of functional importance [59]. Likewise, pseudogenes have the evolutionary potential to become genes with new functions [52,54]. All of these factors indicate that the presence of pseudogenes in genomes is a result of the adaptive genome’s evolution through important regulatory functions that pseudogenes show at the transcriptome level. It is expected that pseudogenes that have positive effects on the whole genome dynamic will be preserved. Today, it is well known that the majority of the genome is transcribed in ncRNA and that some of those transcripts have a role in genome regulation—RNA silencing [56,61].

In the case of *Haus6* gene, difference between sequences of transcripts between samples with and without Bs was not recorded. The transcript of this sequence was present in 50% of animals without Bs, while in the samples with B chromosomes it was present in 85%. *Haus6* gene encodes the HAUS augmin-like protein complex subunit 6, which has a role in microtubule and kinetochore connection and central dividing spindle. This protein has a role in chromosome segregation and drawing to the poles [62]. Since this gene function is directly connected with chromosome distribution in cell division, more frequent expression of this gene in animals with Bs may be directly connected with the mechanism of Bs maintenance in populations. 

For *Cenpe* gene, regarding melting temperature, we did not record any differences in sequences of transcripts between samples with and without Bs, but the level of transcription was significantly statistically higher in the presence of Bs. *Cenpe* gene is coding for a protein that presents a core kinetochore component and has a role as a mediator in connection of microtubules of dividing spindle [63]. A higher transcription level was found in samples with Bs. This difference was more obvious when samples with just one B chromosome were compared with samples without Bs. Nonetheless, comparison of sexes, showed that females with Bs had a three times higher level of transcription while males did not differ significantly. Similarly, a variation in transcription that is connected with the presence of Bs, and a sex-specific phenotype was previously detected for ncDNA repetitive elements originating from Bs in species *A. latifasciata* [28]. Transcription level of these elements was variable depending on tissue type, presence of Bs and sex-specific phenotype in this species. This is not the first case of detection of the absence of Bs transcripts in male individuals in *A. latifasciata*. Carmelo et al. [64] found that the presence of B did not have an impact on males. A link between Bs and sex has been reported for several species [31,65] in a manner that they are more frequent in one of the sexes. In *A. flavicollis*, although there is no difference in Bs appearance and number between sexes, there is a difference in transcriptional activity. The other finding that, in *A. flavicollis,* transcription activity is not proportional to the number of Bs, could indicate some kind of chromosome silencing in a situation when there is more than one B in the genome of an individual. 

Using differential display reverse transcription-polymerase chain reaction (DD RT-PCR), it was previously confirmed in *A. flavicollis* B+ animals higher expression of genes: Chaperonin containing TCP-1, subunit 6b (zeta) (CCT6B), Fragile histidine triad gene (FHIT), and hypothetical gene XP transcript [10]. Those genes affect some of the crucial processes in the cell. It was assumed that Bs with such effects might be advantageous for an organism by increasing tubulin, actin, and other proteins. This indirect evidence, together with herein presented results, point that Bs in this way create an appropriate background for their transmission and maintenance.

Contrary to what used to be a common belief, in this study we found one more species whose sequences on Bs are involved in the transcriptome. Further, B genes, or standard-genome genes in the presence of Bs, show higher transcriptional levels in young females. Additionally, the transcription of some genes could be linked with the number of Bs, in a manner that samples with one B show the highest transcriptional level, while it decreases with a higher number of Bs. One phenomenon observed in early B chromosome research in plants was side-lined in earlier research. In rye and maize and some other plants, it was recorded that Bs behave differently according to whether they were present in odd or even-numbers (reviewed in [2]). This play of even and odd numbers of Bs could also explain differences in their behaviour and effects. Furthermore, Blagojević and Vujošević [37] through studies of the influence of Bs on developmental homeostasis found that carriers of B react differently to environmental changes than do non-carriers. So, according to this study, we could assume, for additional elements in *A. flavicollis* karyotype, that they actively contribute to the transcriptiome and this could depend on sex, age, and the number of Bs. Differential expression of Bs’ genes or genes of the standard genome in the presence of Bs in females indicates the biological significance of these additional elements.

## Figures and Tables

**Table 1:** List of samples used for analyses of transcription activity with data of karyotype, body mass (g), dry eye lens mass (mg) and locality of collection.

Sample	Karyotype	Boody Mass (g)	Eyes Lens Mass (mg)	Locality
3694	48,XX	8.4	12.0	IBISS ^1^
3695	48,XX,+1B	8.1	11.3	IBISS ^1^
3696	48,XY,+1B	9.1	11.4	IBISS ^1^
3698	48,XY	9.9	11.7	IBISS ^1^
3699	48,XY	9.8	12.2	IBISS ^1^
3796	48,XY,+1B	19.8	13.8	Bosilegrad
3860	48,XX,+1B	21.5	17.6	Misača
3907	48,XY,+1B	21.1	15.2	Ruski Krstur
3913	48,XY,+1B	21.4	15.9	Ruski Krstur
4056	48,XX,+1B	18.2	9.9	IBISS ^1^
4060	48,XX	10.0	9.4	IBISS ^1^
4061	48,XY	9.1	8.9	IBISS ^1^
4062	48,XY	10.2	9.0	IBISS ^1^
4063	48,XX	10.3	9.2	IBISS ^1^
4095	48,XX	9.7	8.2	Petnica
4099	48,XX,+1B	10.6	8.2	IBISS ^1^
4112	48,XY,+1B	11.8	8.7	Vlasina
4115	48,XY,+2B	11.4	10.0	Vlasina
4133	48,XX,+1B	12.4	7.5	Babin zub
4134	48,XY	15.6	10.2	Babin zub
4148	48,XX,+1B	32.0	29.9	Petnica
4150	48,XY,+1B	40.6	31.9	Petnica
4157	48,XX,+2B	28.0	30.2	Petnica
4172	48,XX	21.2	17.7	IBISS ^1^
4174	48,XX,+1B	20.6	18.0	IBISS ^1^
4183	48,XX,+2B	31.5	31.1	Maljen
4195	48,XY	17.9	16.2	Goč
4209	48,XY	9.0	7.9	Vlasina
4216	48,XX	19.6	18.9	Vlasina
4218	48,XX	14.5	11.8	Vlasina
4224	48,XX,+2B	17.9	20.8	Vlasina
4227	48,XX,+3B	17.0	18.8	Vlasina
4229	48,XY,+2B	23.7	20.8	Vlasina
4230	48,XY,+1B	18.6	27.2	Vlasina

^1^ offspring of targeted reproduction in population cage.

**Table 2:** Primers used in the study.

Gene		Sequencies
*Rraga*	F	5′GCGGGACAACATCTTCTGTA3′
	R	5′ATCTTTTTCCAGTTCGCGG3′
*Cenpe*	F	5′GAGCCAAGGACTGGCATTAGA3′
	R	5′TGCAGCTTCGATTTGCCTTG3′
*Haus6*	F	5′TTGGCTACAGGGCTCAGTTC3′
	R	5′CTTCCAGAAGAAGTTGGCGA3′
*Calnexin*	F	5′ATGGAAGGGAAGTGGTTACTGT3′
	R	5′GCTTTGTAGGTGACCTTTGGAG3′
*Pgk*	F	5′ATGTCGCTTTCCAACAAGCTG3′
	R	5′GCTCCATTGTCCAAGCAGAAT3′
*β-aktin*	F	5′TGGACATCCGCAAAGACCTGTAC3′
	R	5′TCAGGAGGAGCAATGATCTTGA3′

## Data Availability

Data sharing not applicable.
